# Mathematical analysis of the sodium sensitivity of the human histamine H_3_ receptor

**DOI:** 10.1186/s40203-014-0001-y

**Published:** 2014-05-24

**Authors:** Hans-Joachim Wittmann, Roland Seifert, Andrea Strasser

**Affiliations:** Faculty of Chemistry and Pharmacy, University of Regensburg, Universitätsstraße 31, 93040 Regensburg, Germany; Institute of Pharmacology, Medical School of Hannover, Carl-Neuberg-Straße 1, 30625 Hannover, Germany; Department of Pharmaceutical and Medicinal Chemistry II, Faculty of Chemistry and Pharmacy, University of Regensburg, Universitätsstraße 31, 93040 Regensburg, Germany

**Keywords:** Histamine H_3_ receptor, Na^+^-sensitivity, Mathematical model, Molecular dynamics

## Abstract

**Purpose:**

It was shown by several experimental studies that some G protein coupled receptors (GPCR) are sensitive to sodium ions. Furthermore, mutagenesis studies or the determination of crystal structures of the adenosine A_2A_ or δ-opioid receptor revealed an allosteric Na^+^ binding pocket near to the highly conserved Asp^2.50^. Within a previous study, the influence of NaCl concentration onto the steady-state GTPase activity at the human histamine H_3_ receptor (hH_3_R) in presence of the endogenous histamine or the inverse agonist thioperamide was analyzed. The purpose of the present study was to examine and quantify the Na^+^-sensitivity of hH_3_R on a molecular level.

**Methods:**

To achieve this, we developed a set of equations, describing constitutive activity and the different ligand-receptor equilibria in absence or presence of sodium ions. Furthermore, in order to gain a better understanding of the ligand- and Na^+^-binding to hH_3_R on molecular level, we performed molecular dynamic (MD) simulations.

**Results:**

The analysis of the previously determined experimental steady-state GTPase data with the set of equations presented within this study, reveals that thioperamide binds into the orthosteric binding pocket of the hH_3_R in absence or presence of a Na^+^ in its allosteric binding site. However, the data suggest that thioperamide binds preferentially into the hH_3_R in absence of a sodium ion in its allosteric site. These experimental results were supported by MD simulations of thioperamide in the binding pocket of the inactive hH_3_R. Furthermore, the MD simulations revealed two different binding modes for thioperamide in presence or absence of a Na^+^ in its allosteric site.

**Conclusion:**

The mathematical model presented within this study describes the experimental data regarding the Na^+^-sensitivity of hH_3_R in an excellent manner. Although the present study is focused onto the Na^+^-sensitivity of the hH_3_R, the resulting equations, describing Na^+^- and ligand-binding to a GPCR, can be used for all other ion-sensitive GPCRs.

## Background

G protein coupled receptors (GPCRs), one of the largest protein families within the human genome, play an important role in several physiological and pathophysiological processes (Wise et al. 2002; Foord et al. 2005; Jacoby et al. 2006). In general, the two-state model of GPCR activation suggests that GPCRs can exist in two different states, the inactive “R” and active “R*” state (Schütz and Freissmuth 1992; Lefkowitz et al. 1993; Leff 1995; Seifert and Wenzel-Seifert 2002). These two different conformations were confirmed by crystal structures of the inactive and active state of the β_2_-adrenergic receptor (Cherezov et al. 2007; Rosenbaum et al. 2009; Rasmussen et al. 2011). Ligands, addressing GPCRs, can be classified as inverse agonists, neutral antagonist or agonists (Seifert and Wenzel-Seifert 2002, 2003). It is assumed that inverse agonists stabilize the inactive, whereas agonists stabilize the active state of GPCRs (Seifert and Wenzel-Seifert 2002, 2003). The histamine H_3_ receptor (H_3_R), identified in the early 1980s, is one of four histamine receptor subtypes and belongs to the aminergic GPCRS (Hill et al. 1997; Lovenberg et al. 1999; Leurs et al. 2005; Parsons and Genellin 2006). The H_3_R regulates the release of the endogenous histamine and other neurotransmitters in the nervous system and is involved in important physiological processes, e.g. cognition, eating-behaviour and the sleep-wake cycle (Leurs et al. 2005). For the hH_3_R, a large number of ligands is known (Sasse et al. 2000; Stark et al. 2001; Schnell and Seifert 2010; Strasser et al. 2013; Seifert et al. 2013). However, thioperamide is a standard inverse agonist at the hH_3_R (Arrang et al. 1987; Schnell and Seifert 2010). Some GPCRs, showing constitutive activity, change its conformation from the inactive into the active state in absence of an agonist (Seifert and Wenzel-Seifert 2002, 2003). It was shown that the hH_3_R and the highly related hH_4_R exhibit constitutive activity (Morisset et al. 2000; Schneider et al. 2009; Schnell et al. 2010). Experimental studies revealed that sodium ions can act as an allosteric modulator and stabilize the inactive conformation of a GPCR (Seifert and Wenzel-Seifert 2002, 2003). Experimental studies revealed that only distinct GPCRs are sensitive for sodium ions, whereas other GPCRs are insensitive for sodium ions. The neurotensine receptors (Martin et al. 1999), the D_2_ (Neve 1991; Schetz 2005; Ericksen et al. 2009) for example, are sodium sensitive. Within the family of the histamine receptors, the hH_3_R is sodium sensitive (Schnell and Seifert 2010) whereas the highly related hH_4_R is sodium insensitive (Schneider and Seifert 2009). The corresponding allosteric sodium ion binding site is located between TM II, TM III and TM VII near to the highly conserved Asp^2.50^, as was shown recently with the crystal structure of the human adenosine A_2A_ receptor (hA_2A_R) (Liu et al. 2012) or the δ-opioid receptor (Fenalti et al. 2014). The location of the allosteric sodium binding site was also supported by mutagenesis of the highly conserved Asp^2.50^ into the neutral alanine (Neve et al. 1991; Schetz and Sibley 2001) or asparagine (Ceresa and Limbird 1994; Schnell and Seifert 2010). This is supported by experimental results at hH_3_R, where the Asp^2.50^Asn mutant was found to partially mimic the effect of high sodium chloride concentrations by suppressing constitutive activity (Schnell and Seifert 2010). With MD studies the binding pathway of a sodium ion into the allosteric sodium binding site of the D_2_ receptor (Selent et al. 2010) and the μ-opioid receptor (Yuan et al. 2013) were observed.

**Figure 1:**
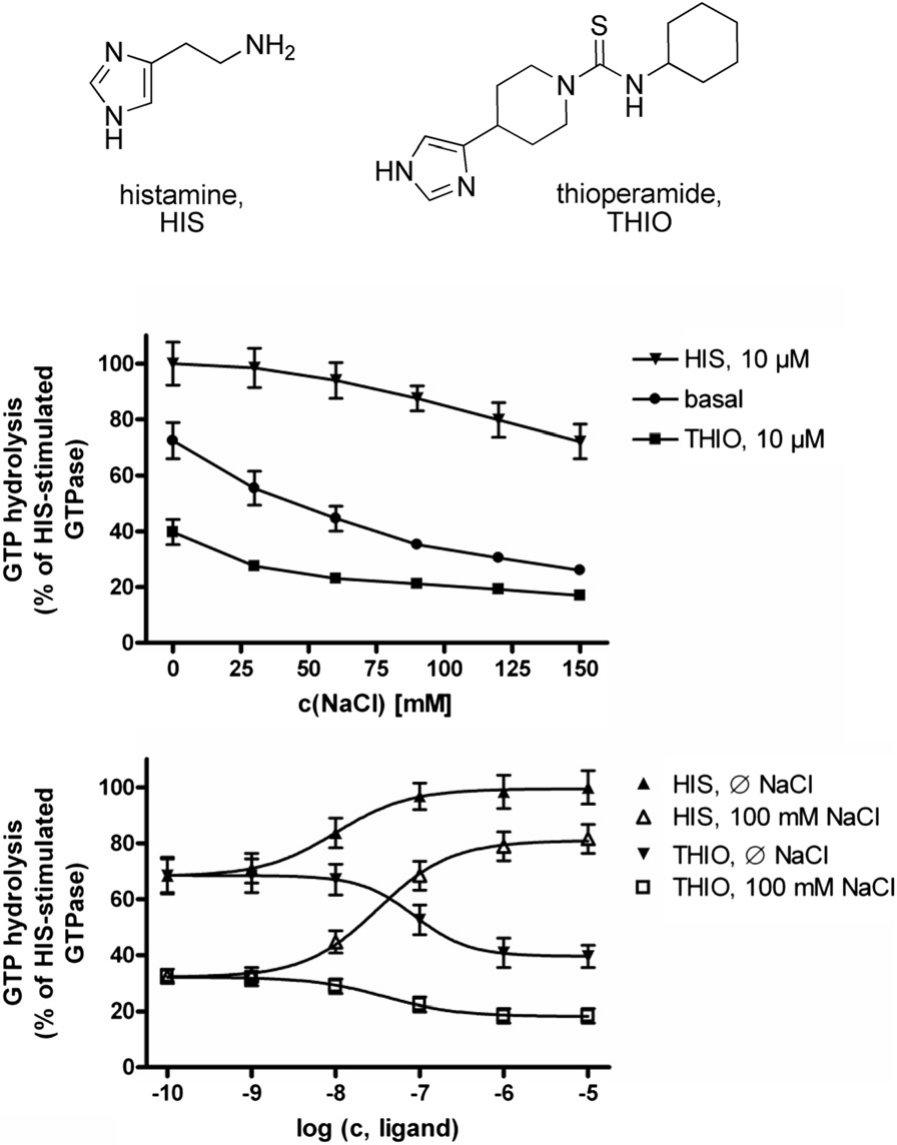
**Sodium sensitivity oh hH**_**3**_ **R. A**, Structures of histamine and thioperamide. **B**, Influence of sodium ions onto the hH_3_R, determined in the steady state GTPase assay. (Figure was adopted from Schnell and Seifert (2010).

During the last decades, several theoretical models were established to explain receptor function quantitatively (Leff 1995; Leff et al. 1997; Christopoulos and Kenakin 2002; Kenakin 2004; Langmead and Christopoulos 2006; Kenakin 2013). Those basic concepts can be extended to describe ion-sensitivity of GPCRs. Within a previous study of Schnell and Seifert (2010), the influence of NaCl concentration onto the concentration response curves of the endogenous histamine and the inverse agonist thioperamide onto the hH_3_R were investigated (Figure 1). Within the present study, we developed a set of equations, describing constitutive activity and the different ligand-receptor equilibra in presence or absence of sodium ions. Furthermore, we used the experimental data, published by Schnell and Seifert (2010) previously (Figure 1) for determination of the equilibrium constants, described by the set of equations, mentioned above, e.g. constants regard to constitutive activity, binding of a sodium ion to the receptor, and binding of histamine or thioperamide to the hH_3_R in absence or presence of sodium ions. Furthermore, MD simulations of several inactive thioperamide-Na^+^-hH_3_R-complexes were performed. These simulations showed that thioperamide can bind into the orthosteric site of hH_3_R in presence or absence of the Na^+^ in the allosteric pocket, resulting in different binding modes of thioperamide.

## Methods

### Equations

For the quantitative description of the sodium sensitivity of the hH_3_R, ten different mass action laws describing the formation of different ligand-receptor complexes were included. Equation (1) takes into account the experimentally observed constitutive activity of the ligand free receptor according to the equilibrium *R* ⇌ R*:

1 $$ {K}_0=\frac{R^{*}}{R} $$

Therein, R* represents the ligand- and sodium free- active receptor and *R* the ligand- and Na^+^- free inactive receptor.

Additionally, distinct Na^+^-receptor equilibriums have to be considered: Equation (2) describes the equilibrium between the inactive receptor *R* and the inactive receptor containing a sodium ion in the orthosteric ligand binding pocket denoted by *A*^*ortho*^*R* according to *A* + *R* ⇌ *A*^*ortho*^*R*. Therein, the concentration of sodium chloride *A* is considered to be equal to the overall concentration *A*_*0*_ of sodium chloride, because of the much smaller concentration of the receptor species. This approximation holds also for the ligands thioperamide (*B*) and histamine (*C*).

2 $$ {K}_1=\frac{A^{ortho}R}{A_o\cdot R} $$

In general, it has to be taken into account (equation 3) that sodium ions are able to bind into the orthosteric binding pocket of the active receptor establishing the complex *A*^*ortho*^R* according to *A* + R* ⇌ *A*^*ortho*^R*:

3 $$ {K}_2=\frac{A^{ortho}{R}^{*}}{A_0\cdot {R}^{*}} $$

Based on experimental studies, it is suggested that a sodium ion *A* can bind from its orthosteric into its allosteric binding pocket to form the species *A*^*allo*^*R* according to *A*^*ortho*^*R* ⇌ *A*^*allo*^*R*. This fact is considered in equation 4.

4 $$ {K}_3=\frac{A^{allo}R}{A^{ortho}R} $$

Additionally in the presence of thioperamide marked by *B*, the following equations (5, 6, 7) have to be considered. Thioperamide *B* can bind into the orthosteric ligand binding site of the inactive receptor and a corresponding thioperamide-receptor complex *BR* is formed (equation 5) according to *B* + *R* ⇌ *BR*.

5 $$ {K}_4=\frac{ BR}{B_0\cdot R} $$

There, *B*_*0*_ represents the total concentration of thioperamide. Furthermore, it has to be taken into account that thioperamide may also be able to bind into the orthosteric ligand binding site of the active receptor (equation 6) and a corresponding active thioperamide-receptor complex BR* is formed according to *B* + R* ⇌ BR^*^.

6 $$ {K}_5=\frac{{ BR}^{*}}{B_0\cdot {R}^{*}} $$

Next, it has to be considered that thioperamide binds into the orthosteric binding site when a sodium ion is bound into the allosteric binding site of the inactive receptor (equation 7) forming the complex *BA*^*allo*^*R* according to *B* + *A*^*allo*^*R* ⇌ *BA*^*allo*^*R*.

7 $$ {K}_6=\frac{B{A}^{allo}R}{B_0\cdot {A}^{allo}R} $$

In case that histamine *C* is present, the equations (8, 9, 10) have to be taken into account. In general it should be considered that histamine *C* is able to bind into the orthosteric ligand binding site of the inactive receptor (equation 8) and a corresponding histamine-receptor complex *CR* is formed according to *C* + *R* ⇌ *CR*:

8 $$ {K}_7=\frac{ CR}{C_0\cdot R} $$

where *C*_*0*_ describes the total concentration of histamine. Furthermore, it has to be taken into account that histamine binds into the orthosteric ligand binding site of the active receptor (equation 9) and a corresponding active histamine-receptor complex CR* is formed according to *C* + R* ⇌ CR^*^:

9 $$ {K}_8=\frac{{ CR}^{*}}{C_0\cdot {R}^{*}} $$

Additionally, it has to be expected that histamine binds into the orthosteric binding site when a sodium ion is bound into the allosteric binding site (equation 10) of the inactive receptor *CA*^*allo*^*R* at the same time according to *C* + *A*^*allo*^*R* ⇌ *CA*^*allo*^*R*:

10 $$ {K}_9=\frac{C{A}^{allo}R}{C_0\cdot {A}^{allo}R} $$

It is suggested that sodium ions are not able to bind into the allosteric binding site of the active receptor (Liu et al. 2012). Therefore, the corresponding complexes are not considered within the presented system of equations. Once more, it should be mentioned that the overall concentrations of sodium chloride, thioperamide and histamine (eqs. 2, 3, 4, 5, 6, 7, 8, 9 and 10) are much higher than the total concentration of the receptor, so no change of that quantities will be detected when reaching the thermodynamic equilibrium. Equation (11) results from the law of conservation of matter referring to the receptor.

11 $$ {R}_0=R+{R}^{*}+{A}^{ortho}R+{A}^{ortho}{R}^{*}+{A}^{allo}R+ BR+{ BR}^{*}+B{A}^{allo}R+ CR+{ CR}^{*}+C{A}^{allo}R $$

The solution of the equations 1, 2, 3, 4, 5, 6, 7, 8, 9, 10 and 11 with respect to the concentration terms was calculated using the software package MAPLE 11.0 (Maplesoft Waterloo Maple Inc. 1981-2007). Maple is a computer algebra system, allowing users to define mathematical equations in a simple manner, solve these equations with one command line and plot the corresponding results. To elucidate this, the definition of equations (1, 2, 3, 4, 5, 6, 7, 8, 9, 10 and 11) (see the following command lines e1 to e11) and their solution with the help of the command “solve” (see command line e12) is shown, using the Maple syntax:

- > e1:=K0 = RS/R:
- > e2:=K1 = AR/(A0*R):
- > e3:=K2 = ARS/(A0*RS):
- > e4:=K3 = ARR/AR:
- > e5:=K4 = BR/(B0*R):
- > e6:=K5 = BRS/(B0*RS):
- > e7:=K6 = BRR/(B0*ARR):
- > e8:=K7 = CR/(C0*R):
- > e9:=K8 = CRS/(C0*RS):
- > e10:=K9 = CRR/(C0*ARR):
- > e11:=R0 = R + RS + AR + ARS + ARR + BR + BRS + BRR + CR + CRS + CRR:
- > e12:=solve({e1,e2,e3,e4,e5,e6,e7,e8,e9,e10,e11},{R,RS,AR,ARS,ARR,BR,BRS,BRR,CR,CRS,CRR});

The last command led to the following expressions for the concentration terms (Maple notation in brackets) *R* (R), R* (RS), *A*^*ortho*^*R* (AR), *A*^*ortho*^R* (ARS), *A*^*allo*^*R* (ARR), *BR* (BR), BR* (BRS), *BA*^*allo*^*R* (BRR), *CR* (CR), CR* (CRS) and *CA*^*allo*^*R* (CRR):

The denominator *d* of the following terms reads as:

12 $$ \begin{array}{l}d=1+{K}_0+{K}_1{A}_0+{K}_4{B}_0+{K}_7{C}_0+{K}_0{K}_2{A}_0+{K}_1{K}_3{A}_0+{K}_0{K}_5{B}_0+{K}_0{K}_8{C}_0+\\ {}{K}_1{K}_3{K}_6{A}_0{B}_0+{K}_1{K}_3{K}_9{A}_0{C}_0\end{array} $$

13 $$ R=\frac{R_0}{d} $$

14 $$ R*=\frac{K_0{R}_0}{d} $$

15 $$ {A}^{ortho}R=\frac{K_1{A}_0{R}_0}{d} $$

16 $$ {A}^{ortho}R*=\frac{K_0{K}_2{A}_0{R}_0}{d} $$

17 $$ {A}^{allo}R=\frac{K_1{K}_3{A}_0{R}_0}{d} $$

18 $$ BR=\frac{K_4{B}_0{R}_0}{d} $$

19 $$ BR*=\frac{K_0{K}_5{B}_0{R}_0}{d} $$

20 $$ B{A}^{allo}R=\frac{K_1{K}_3{K}_6{A}_0{B}_0{R}_0}{d} $$

21 $$ CR=\frac{K_7{C}_0{R}_0}{d} $$

22 $$ CR*=\frac{K_0{K}_8{C}_0{R}_0}{d} $$

23 $$ C{A}^{allo}R=\frac{K_1{K}_3{K}_9{A}_0{C}_0{R}_0}{d} $$

### Description of the efficacy

The efficacy determined by steady-state GTPase assay is assumed to be proportional (described by factor *f*, assuming that *f* is independent of any concentration) to all complexes containing an active receptor configuration (equation 24):

24 $$ E=f\cdot \left({R}^{*}+{ AR}^{*}+{ BR}^{*}+{ CR}^{*}\right) $$

To introduce the constants *K*_*0*_ to *K*_*9*_ in equation 24, the equations 14, 16, 19 and 22 can be introduced into 24, leading to the following equation 25:

25 $$ E=\frac{f{R}_0{K}_0\left(1+{K}_2{A}_0+{K}_5{B}_0+{K}_8{C}_0\right)}{d} $$

The application of the present mathematical concept onto experimental data values requires to define a reference value *E*_*ref*_ of the effect *E*: *E*_*ref*_ (equation 26) represents the effect at *A*_*0*_ = *B*_*0*_ = 0 and *C*_*0*_^*ref*^ = 10 μM and is obtained from equation 25:

26 $$ {E}_{ref}=\frac{f{R}_0{K}_0\left(1+{K}_8{C}_0^{ref}\right)}{1+{K}_0+{K}_7{C}_0^{ref}+{K}_0{K}_8{C}_0^{ref}} $$

The resulting relative quantity *E*_*rel*_ (equation 27) corresponds directly to the experimental data (Schnell and Seifert 2010) shown in Figure 1.

27 $$ {E}_{rel}=\frac{E}{E_{ref}}=\frac{\left(1+{K}_2{A}_0+{K}_5{B}_0+{K}_8{C}_0\right)\left(1+{K}_0{K}_7{C}_0^{ref}+{K}_0{K}_8{C}_0^{ref}\right)}{d\cdot \left(1+{K}_8{C}_0^{ref}\right)} $$

Thus, if only thioperamide (B) and sodium ions (A) are present, equation 27 can be written as

28 $$ {E}_{rel}=\frac{\left(1+{K}_2{A}_0+{K}_5{B}_0\right)\left(1+{K}_0{K}_7{C}_0^{ref}+{K}_0{K}_8{C}_0^{ref}\right)}{\left(1+{K}_0+{K}_1{A}_0+{K}_4{B}_0+{K}_0{K}_2{A}_0+{K}_1{K}_3{A}_0+{K}_0{K}_5{B}_0+{K}_1{K}_3{K}_6{A}_0{B}_0\right)\left(1+{K}_8{C}_0^{ref}\right)} $$

and if only histamine (C) and sodium ions (A) are present, as

29 $$ {E}_{rel}=\frac{\left(1+{K}_2{A}_0+{K}_8{C}_0\right)\left(1+{K}_0{K}_7{C}_0^{ref}+{K}_0{K}_8{C}_0^{ref}\right)}{\left(1+{K}_0+{K}_1{A}_0+{K}_7{C}_0+{K}_0{K}_2{A}_0+{K}_1{K}_3{A}_0+{K}_0{K}_8{C}_0+{K}_1{K}_3{K}_9{A}_0{C}_0\right)\left(1+{K}_8{C}_0^{ref}\right)} $$

### Least-square fit to obtain the constants *K*_*x*_

To determine the constants *K*_*x*_ (*K*_*0*_ to *K*_*9*_) the software MAPLE 11.0 was used. For fitting of the experimental data (Schnell and Seifert 2010), equation 27 was used, where the constants *K*_*x*_ were substituted by *K*_*x*_ *= 10*^*pKx*^. The least-square fit is based on the following equation 30, describing the deviance s^2^:

30 $$ {s}^2={\displaystyle \sum_i{\left({E}_{\exp, i}^{rel}-{E}_{calc,i}^{rel}\right)}^2} $$

There, ${E}_{\exp, i}^{rel}$ represents each experimentally determined data point *i* shown in Figure 1, relative to the effect, determined at a histamine concentration of *C*_*0*_^*ref*^ = 10 μM in absence of sodium chloride and thioperamide. ${E}_{calc,i}^{rel}$ represents the calculated relative effect according to equation 25 for a set of constants *K*_*x*_ to be determined by searching the minimum of *s*^*2*^ using the software MAPLE 11.0. However, to solve this problem, every other software package can be used.

### Construction of the inactive model of hH_3_R

For the construction of the homology model of the inactive hH_3_R, the crystal structure of the inactive hH_1_R (3RZE) (Shimamura et al. 2011) was used as a template. The hH_3_R homology model was designed using SYBYL 7.0 (Tripos; http://www.tripos.com) according to a protocol, described previously (Strasser and Wittmann 2013; Darras et al. 2014; Wagner et al. 2014). Briefly, the artificial lysozyme in 3RZE was deleted and the homology model was generated according to a hH_1_R-hH_3_R amino acid alignment already described (Strasser et al. 2013). The N-terminus, missing in the crystal structure of hH_1_R, was completed using SYBYL 7.0 (Tripos Inc), as described previously (Darras et al. 2014; Wagner et al. 2014). Furthermore, the E2-loop was completed using the “Loop-Search” module of SYBYL 7.0, as described previously for the hH_4_R (Darras et al. 2014; Wagner et al. 2014). Because there is no information about the conformation of the long I3-loop of the hH_3_R, containing more than 100 amino acids, the amino acids Ala^239^ to Arg^347^ were not included into the model. However, to close the resulting gap between TM V and TM VI on the intracellular side, eight alanines were inserted instead. It was shown previously, that internal water molecules play an important role in stabilization or activation of aminergic GPCRs (Angel et al. 2009; Liu et al. 2012). Therefore, the internal water molecules, described in literature were included according to the corresponding crystal structures (Angel et al. 2009; Liu et al. 2012). In a first model, thioperamide was docked manually into the binding pocket of hH_3_R, in such manner that the positively charged imidazole moiety interacts electrostatically with the highly conserved Asp^3.32^, according to the binding mode for analogue compounds, already described in literature (Schnell and Seifert 2010). The remaining part of thioperamide was embedded in a pocket between TM III, TM V and TM VI, as already described for similar H_3_ receptor ligands (Schlegel et al. 2007; Schnell and Seifert 2010). In a second model, one sodium ion was docked manually into the allosteric binding site of hH_3_R, according to the crystal structure of the A_2A_ with a Na^+^ in the allosteric binding site (Liu et al. 2012). Furthermore, a third model, containing thioperamide in the orthosteric and one sodium ion in the allosteric binding site was constructed, as described above for the thioperamide- and the sodium-ion- model. The resulting complexes were minimized energetically with SYBYL 7.0. Subsequently the minimized hH_3_R-models containing thioperamide and/or a sodium ion were embedded in a POPC lipid bilayer. Afterwards, intracellular and extracellular water molecules were added. To achieve electroneutrality, an appropriate number of sodium ions and chloride ions were added into the simulation box. Subsequently, MD simulations were performed with GROMACS 4.0.2 (http://www.gromacs.org) as already described (Strasser et al. 2008; Igel et al. 2009; Darras et al. 2014). The parameterization for thioperamide was obtained from the PRODRG server (http://davapc1.bioch.dundee.ac.uk/prodrg/). However, the partial charges were adopted by the Gasteiger-Hückel partial charged, calculated with SYBYL 7.0. The force field parameters for the POPC lipids were obtained from the online source http://moose.bio.ucalgary.ca/index.php?page=Structures_and_Topologies. For equilibration, a 5 ns MD simulation was performed: Within the first 2.5 ns, force constants of 250 kJ/(mol nm^2^) were put onto the backbone atoms of the TM domains of hH_3_R, within the second 2.5 ns, these force constants were reduced to 100 kJ/(mol nm^2^). Subsequently, 10 ns up to 35 ns productive phase of simulations were performed, without using any force constants.

## Results

### Constants *K*_*0*_ to *K*_*9*_ for the hH_3_R, determined by steady-state GTPase assays

The nonlinear least square fit was used to determine the constants *K*_*0*_ to *K*_*9*_ (*K*_*x*_), expressed as the corresponding values *pK*_*0*_ – *pK*_*9*_ (*pK*_*x*_) , using the definition *K*_*x*_ *= 10*^*pKx*^, as described under Materials and Methods (Table 1).

**Table 1:** **pK**_**x**_ **, K**_**x**_ **and Δ**_**R**_ **G** ^**o**^ **values, describing the constitutive activity, the binding of sodium ions, of thioperamide and histamine to hH**_**3**_ **R**

	**x**		***pK***_***x***_ **± S.E.M.**	***K***_***x***_ **± S.E.M.**	***Δ***_***R***_ ***G*** ^***o***^ **[kJ/mol]**
Constitutive activity	0	*R* ⇌ R*	-0.04 ± 0.01	0.92 ± 0.03	0.21 ± 0.08
NaCl (≡A)	1	*A* + *R* ⇌ *A* ^*ortho*^ *R*	0.31 ± 0.03	2.08 ± 0.20	-1.76 ± 0.20
	2	*A* + R* ⇌ *A* ^*ortho*^ R*	0.06 ± 0.08	1.29 ± 0.22	-0.33 ± 0.47
	3	*A* ^*ortho*^ *R* ⇌ *A* ^*allo*^ *R*	1.07 ± 0.04	11.98 ± 0.84	-6.10 ± 0.21
THIO (≡B)	4	*B* + *R* ⇌ *BR*	7.40 ± 0.03	2.56 · 10^7^ ± 1.35 · 10^6^	-42.25 ± 0.15
	5	*B* + R* ⇌ *BR* ^*^	7.01 ± 0.03	1.03 · 10^7^ ± 6.15 · 10^5^	-39.98 ± 0.17
	6	*B* + *A* ^*allo*^ *R* ⇌ *BA* ^*allo*^ *R*	7.23 ± 0.03	1.71 · 10^7^ ± 9.96 · 10^5^	-41.25 ± 0.15
HIS (≡C)	7	*C* + *R* ⇌ *CR*	7.48 ± 0.01	3.05 · 10^7^ ± 1.07 · 10^6^	-42.71 ± 0.08
	8	*C* + R* ⇌ *CR* ^*^	7.86 ± 0.01	7.20 · 10^7^ ± 1.71 · 10^6^	-44.84 ± 0.06
	9	*C* + *A* ^*allo*^ *R* ⇌ *CA* ^*allo*^ *R*	6.98 ± 0.01	9.48 · 10^6^ ± 3.02 · 10^5^	-39.81 ± 0.08

The data were obtained by a least-square fit of experimental data (Schnell and Seifert 2010), as described under Materials and Methods.

The constant *K*_*0*_, describing the constitutive activity has a value of 0.92. Thus, the active hH_3_R (R*) is decreased in stability of *Δ*_*R*_*G*^*o*^ = 0.21 kJ/mol compared to the inactive hH_3_R (*R*) (Table 1) according to *R* ⇌ R*.

The binding of a sodium ion from the aqueous extracellular side into the allosteric binding site can be divided into two steps, according to the equations 2 and 4. The binding of the Na^+^ into the orthosteric binding site (*A*^*ortho*^*R*) according to *A* + *R* ⇌ *A*^*ortho*^*R* with an association constant *K*_*1*_ of 2.08 is energetically favoured (*Δ*_*R*_*G*^*o*^ = -1.76 kJ/mol) (Table 1). The subsequent binding of the sodium ion from the orthosteric into the allosteric binding site, with a *K*_*3*_ of 11.98, according to *A*^*ortho*^*R* ⇌ *A*^*allo*^*R*, is energetically favoured with *Δ*_*R*_*G*^*o*^ = -6.10 kJ/mol (Table 1). Thus, the consecutive binding process of the sodium ion into its allosteric binding site, according to *A* + *R* ⇌ *A*^*ortho*^*R*, is energetically favoured with *Δ*_*R*_*G*^*o*^ = -7.86 kJ/mol. The binding of a sodium ion into the orthosteric binding site of the active hH_3_R, according to *A* + R* ⇌ *A*^*ortho*^R*, with *Δ*_*R*_*G*^*o*^ = -0.33 kJ/mol does not differ significantly from zero (Table 1).

The binding of thioperamide to the orthosteric binding site of hH_3_R in absence of a sodium ion in the allosteric binding site is preferred compared to the binding in presence of a sodium ion in the allosteric binding site, as indicated by the corresponding association constants *K*_*4*_, according to *B* + *R* ⇌ *BR*, and *K*_*6*_, according to *B* + *A*^*allo*^*R* ⇌ *BA*^*allo*^*R*. The association constant *K*_*5*_ for the binding of thioperamide to the active state hH_3_R, according to *B* + R* ⇌ BR^*^ is smaller than *K*_*4*_ or *K*_*6*_ (Table 1), which is in good accordance to the experimental findings that thioperamide acts as an inverse agonist at hH_3_R (Schnell and Seifert 2010).

**Figure 2:**
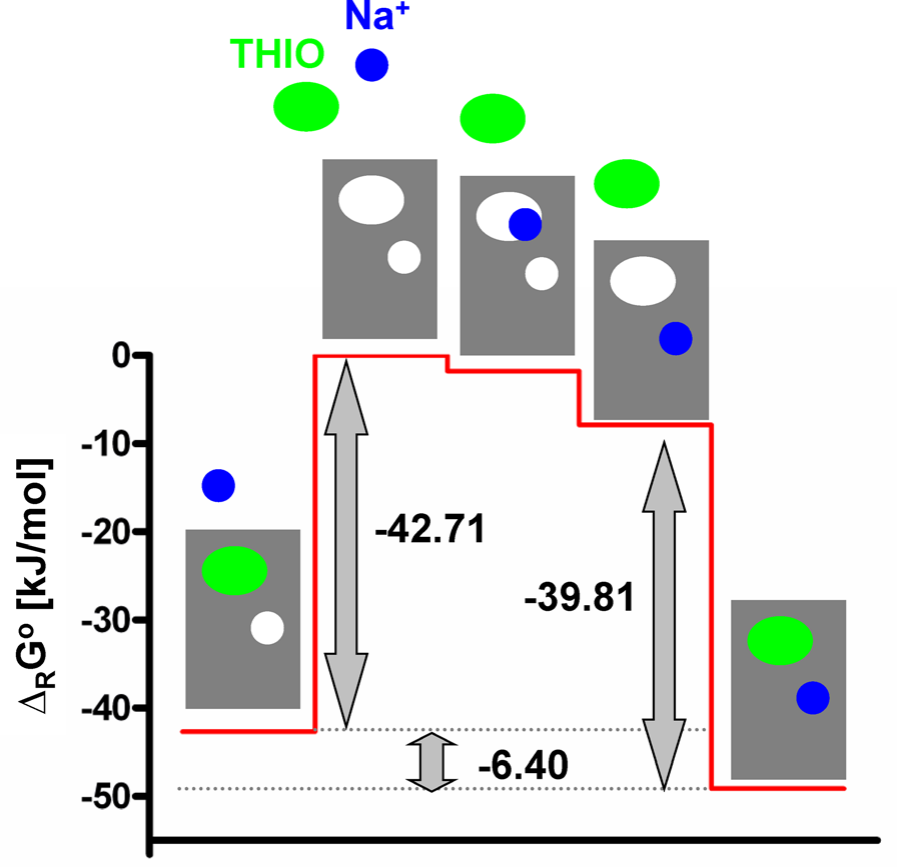
**Gibbs energies (Δ**_**R**_ **G** ^**O**^ **) according to the constants K**_**x**_ **, obtained by fitting experimental data.** Schematic Gibbs energy (*Δ*_*R*_ *G* ^*O*^) profile for the processes *A + B + R ⇌ A + BR* (left branch) and *A + B + R ⇌* B + *A* ^*ortho*^ *R ⇌ B + A* ^*allo*^ *R ⇌ BA* ^*allo*^ *R* (right branch) based on the experimentally determined *K*_*x*_ values (Table 1).

To explain the different amounts of *BR* and *BA*^*allo*^*R* Figure 2 represents the Gibbs energy (*Δ*_*R*_*G*^*o*^) profile for the processes *A + B + R ⇌ A + BR* and *B + A*^*allo*^*R⇌ BA*^*allo*^*R*. The quantity *Δ*_*R*_*G*^*o*^ for the latter process is disfavoured compared to the first one. Nevertheless, the concentration of the complex *BA*^*allo*^R is higher than that of the complex *BR*. The reason for this fact are the two preceding processes *A + B + R ⇌ B + A*^*ortho*^*R* and B + *A*^*ortho*^*R ⇌ B + A*^*allo*^*R* resulting in a stabilization of the complex *BA*^*allo*^*R*, representing thioperamide in the inactive hH_3_R with Na^+^ present in its allosteric binding site (Figure 2).

The association constant *K*_*8*_ of the endogenous agonist histamine to the active hH_3_R, according to *C* + R* ⇌ CR^*^, is higher than for the binding to the inactive hH_3_R in absence (*K*_*7*_) or presence (*K*_*9*_) of a sodium ion in the allosteric binding site (Table 1). This is in good accordance to the experimental findings revealing histamine as an agonist at hH_3_R (Schnell and Seifert 2010).

### Relative concentration profiles of different receptor complexes in dependence of thioperamide, histamine and NaCl in the steady-state GTPase assay at hH_3_R

**Figure 3:**
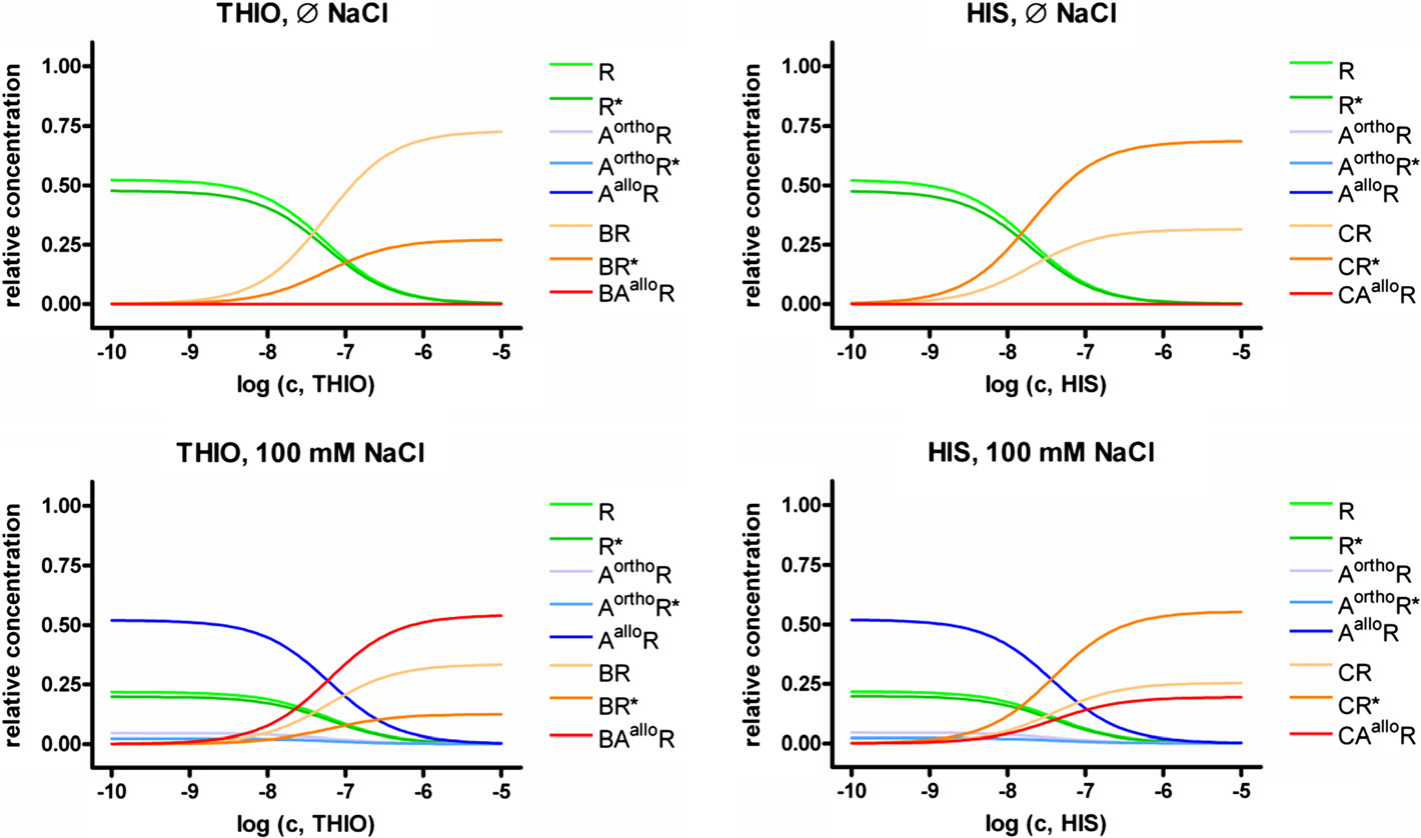
**Concentration profiles of different Na** ^**+**^ **- or ligand-hH**_**3**_ **R complexes in absence or presence of NaCl.** The concentration profiles were calculated according to the equations 13, 14, 15, 16, 17, 18, 19, 20, 21, 22 and 23, using the constants *K*_*x*_ (Table 1), obtained by fitting of the experimental data of Schnell and Seifert 2010, shown in Figure 1, as described in Materials and Methods.

The relative concentration profiles (Figure 3) of the different species mentioned above, as function of concentrations of thioperamide, histamine and sodium chloride were calculated based on the association constants, given in Table 1. In absence or presence of 100 mM NaCl, the amount of the ligand- and Na^+^-free receptor states R* and *R* decreases to zero with increasing concentration of thioperamide (Figure 3). The amount of *BR* (inactive thioperamide-hH_3_R-complex without Na^+^) and BR* (active thioperamide-hH_3_R-complex without Na^+^) increases in absence of sodium chloride with increasing concentration of thioperamide. However, the concentration of BR* is smaller than of *BR*, which is in good accordance to the experimental findings that thioperamide is an inverse agonist (Schnell and Seifert 2010). Based on these data it has to be suggested that thioperamide binds not only to the inactive hH_3_R but also to the active state hH_3_R (Table 1). Consequently, thioperamide has to be defined as a partial inverse agonist. To support these results, analogous calculations, but without including equation 6 and related variables were not able to fit the experimental data. With increasing concentrations of sodium chloride, the amount of *BA*^*allo*^*R* increases, whereas the amount of *BR* decreases. At a concentration of about 62 mM, the relative concentrations of *BR* and *BA*^*allo*^*R* are identical. Thus, at concentrations of sodium chloride < 62 mM, the relative concentration of *BR* is larger than of *BA*^*allo*^*R*, whereas at concentrations of sodium chloride > 62 mM, the relative concentration of *BR* is smaller than of *BA*^*allo*^*R*. The latter case is presented for a concentration of sodium chloride of 100 mM in Figure 3: There, the amount of *BA*^*allo*^*R* is about factor 1.5 higher than the amount of *BR*.

In absence or presence of 100 mM NaCl, the amount of the ligand- and Na^+^-free receptor states R* and *R* decreases to zero with increasing concentrations of histamine whereas the amount of CR* increases (Figure 3). However, regardless of the sodium chloride concentration, a small amount of the Na^+^-free inactive histamine-hH_3_R-complex (*CR*) is present. With increasing concentration of sodium chloride, the amount of *CA*^*allo*^*R* increases. At a concentration of about 285 mM of NaCl, the concentrations of CR* and *CA*^*allo*^*R* are nearly equal. For all concentrations of sodium chloride less than 285 mM, CR* is higher than *CA*^*allo*^*R* (Figure 3). Based on these data it has to be suggested that histamine binds also in the inactive state hH_3_R with or without Na^+^ bound in its allosteric binding site. However, histamine is defined as a full agonist, because there is no other ligand with higher stimulatory effect than histamine at hH_3_R. To support these results, analogue calculations, but without including equations 8 and/or 10 and related variables were performed, but were not able to fit the experimental data. However, in presence of 100 mM NaCl, the concentration of CR* is about 3 times higher than that of *CA*^*allo*^*R* (Figure 3).

### Explanation of the steady-state GTPase results for the hH_3_R

**Figure 4:**
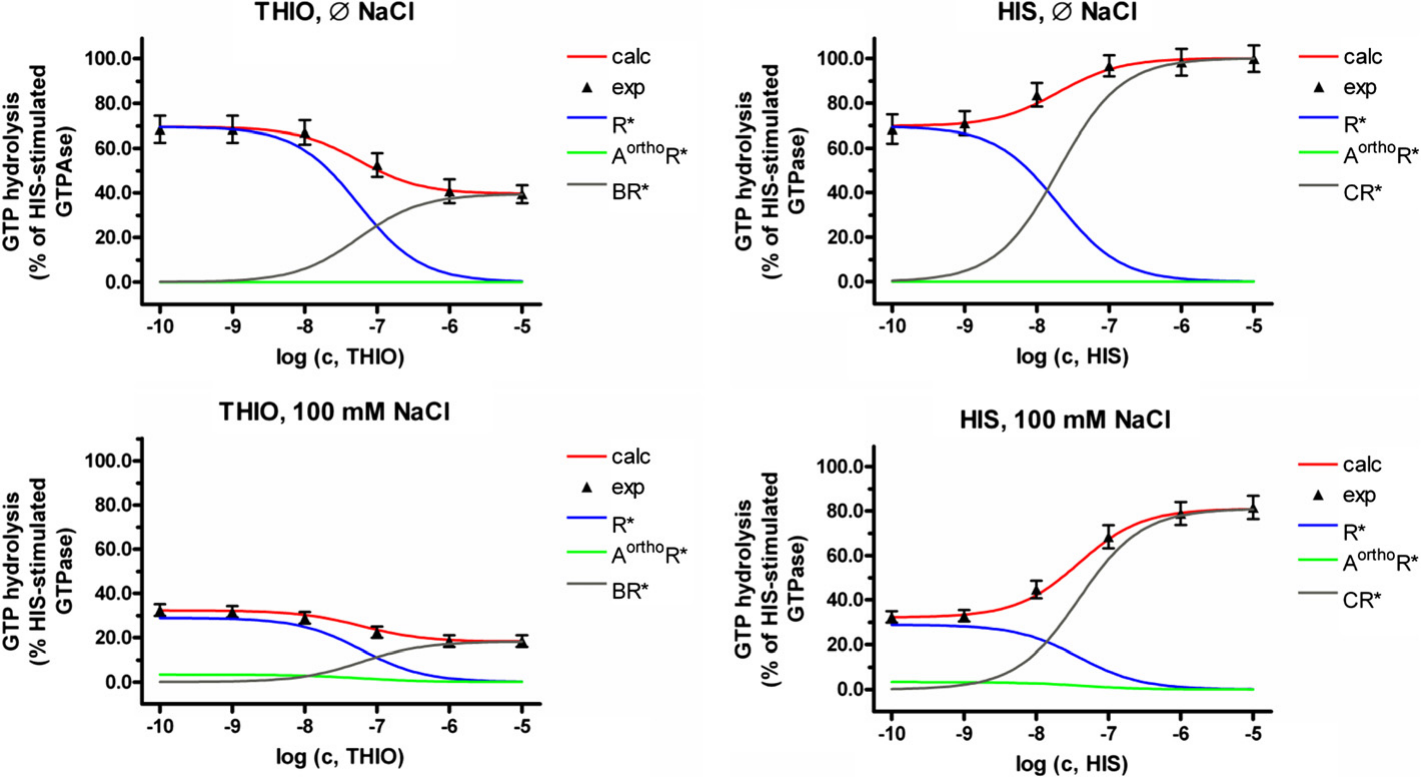
**Portion of** R* **,** ***A*** ^***ortho***^ R* **and** BR* **or** CR* **, in dependence of ligand concentration in GTP hydrolysis and the resulting steady-state GTPase course.** The profiles were calculated according to the equations 14, 16, 19, 23, 24 and 26 using the constants *K*_*x*_ (Table 1), obtained by fitting of the experimental data of Schnell and Seifert 2010, shown in Figure 1, as described in Materials and Methods.

**Figure 5:**
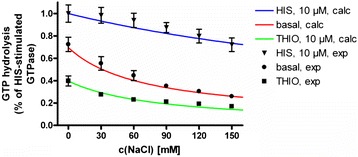
**Portion of** R* **,** ***A*** ^***ortho***^ R* **and** BR* **or** CR* **, in dependence of NaCl concentration in GTP hydrolysis and the resulting steady-state GTPase course.** The profiles were calculated according to equation 27 using the constants *K*_*x*_ (Table 1), obtained by fitting of the experimental data of Schnell and Seifert 2010, shown in Figure 1, as described in Materials and Methods.

In general, it is suggested that only active hH_3_R complexes induce GTPase activity. Thus, to explain the results of the steady-state GTPase assay at hH_3_R, the complexes R*, *A*^*ortho*^R* and BR* in case of thioperamide and R*, *A*^*ortho*^R* and CR* in case of histamine have to be considered. The sum of these values results in the experimentally determined course of GTPase activity (Figures 4 and 5). Figure 4 shows that the experimentally observed courses within the steady-state GTPase assay for thioperamide or histamine in absence or presence of 100 mM NaCl at hH_3_R, are the sum of at least two different courses, namely R* and BR* (in case of thioperamide) and R* and CR* (in case of histamine). The third complex, *A*^*ortho*^R*, is zero in absence of NaCl or nearly zero in presence of 100 mM NaCl. The data for thioperamide (Figure 4) show that the contribution of R* to the GTPase activity decreases with increasing thioperamide concentration. In contrast, the contribution of the active thioperamide-hH_3_R-complexes (BR*) to the GTPase activity increases (Figure 4). But because the increase of BR* is not as strong as the decrease of R*, the final GTPase activity is decreased with increasing thioperamide concentration, which is in very good accordance to experimental results (Schnell and Seifert 2010). The data for histamine (Figure 4) show that the contribution of R* to the GTPase activity decreases with increasing histamine concentration. In contrast, the contribution of the active histamine-hH_3_R-complexes (CR*) to the GTPase activity increases (Figure 4). Because the increase of CR* is stronger, as the decrease of R*, the final GTPase activity is increased with increasing histamine concentration, which is in very good accordance to experimental results (Schnell and Seifert 2010). As provided by Figure 5, the calculated course of basal GTPase activity or GTPase activity in presence of 10 μM histamine or thioperamide in dependence of NaCl concentration is in very good accordance to the experimental data.

### Calculation of the *pEC*_*50*_ values

Additionally, it is possible to deduce an equation for the experimentally observed *pEC*_*50*_ value from the equations (28 and 29) for thioperamide in dependence of the sodium ion concentration *A*_*0*_:

31 $$ pE{C}_{50}=- \log \left(\frac{1+{K}_0+{A}_0\left({K}_1+{K}_0{K}_2+{K}_1{K}_3\right)}{K_4+{K}_0{K}_5+{A}_0{K}_1{K}_3{K}_6}\right) $$

and analogously for histamine:

32 $$ pE{C}_{50}=- \log \left(\frac{1+{K}_0+{A}_0\left({K}_1+{K}_0{K}_2+{K}_1{K}_3\right)}{K_7+{K}_0{K}_8+{A}_0{K}_1{K}_3{K}_9}\right) $$

These equations show that the *pEC*_*50*_ value is dependent of the amount of constitutive activity, described by *K*_*0*_ as well as of the constants *K*_*1*_, *K*_*2*_ and *K*_*3*_, describing the three different equilibria between sodium ions and the receptor (equations 2, 3, 4), and the concentration of sodium ion *A*_*0*_ itself. Furthermore, the ligand specific constants (thioperamide: *K*_*4*_, *K*_*5*_ and *K*_*6*_; histamine: *K*_*7*_, *K*_*8*_ and *K*_*9*_) have an influence onto the *pEC*_*50*_. The equations show that the *pEC*_*50*_ increases with increase of the ligand-receptor specific constants *K*_*4*_, *K*_*5*_, *K*_*6*_ (thioperamide) or *K*_*7*_, *K*_*8*_, *K*_*9*_ (histamine). Furthermore, the *pEC*_*50*_ increases with decreasing *K*_*2*_. If no sodium chloride is present, the constants *K*_*1*_, *K*_*2*_, *K*_*3*_, *K*_*6*_ (thioperamide) and *K*_*9*_ (histamine) are not relevant. Because of the complexity of the equations 31 and 32, the influence of *K*_*0*_, *K*_*1*_, *K*_*3*_ and *A*_*0*_ onto the *pEC*_*50*_ depends on the values of the other variables in the equations, and thus, no simple rules to describe the influence of each *K*_*x*_ onto *pEC*_*50*_ can be presented.

Using the *K*_*x*_ values, obtained by the fit of the experimental data (Table 1), for the GTPase curve of thioperamide in absence of NaCl, a *pEC*_*50*_ of 7.26 and in presence of 100 mM NaCl, a *pEC*_*50*_ of 7.21 was obtained. The *pEC*_*50*_ value in absence of sodium chloride fits well to the experimental data (7.15 ± 0.31 (Schnell and Seifert 2010)). The calculated *pEC*_*50*_ value in presence of 100 mM NaCl does not differ within the limits of error from the experimental data (7.43 ± 0.28 (Schnell and Seifert 2010)). Using the *K*_*x*_ values, obtained by the fit of the experimental data (Table 1), for the GTPase curve of histamine in absence of NaCl, a *pEC*_*50*_ of 7.70 and in presence of 100 mM NaCl, a *pEC*_*50*_ of 7.41 was obtained. The *pEC*_*50*_ value in absence (exp.: 8.01 ± 0.39 (Schnell and Seifert 2010)) and presence (exp.: 7.53 ± 0.18 (Schnell and Seifert 2010)) of sodium chloride fit well to the experimental data.

### Molecular dynamics of different thioperamide- and Na^+^-hH_3_R-complexes

In order to study the influence of a sodium ion in its allosteric binding site onto the conformation of the ligand-free inactive hH_3_R, two different MD simulations were performed: On the one hand, one Na^+^ was placed into its allosteric site, according to the crystal structures of the A_2A_ (Liu et al. 2012). For purpose of reference, an identical system, except with the Na^+^ not located in the allosteric binding site of the hH_3_R, but somewhere in the aqueous extracellular part of the simulation box was built. The MD simulations, performed under comparable conditions, revealed a stabilization of the inactive conformation of the hH_3_R, with the sodium ion being stable in its allosteric binding site. In contrast for the reference system, without a Na^+^ in the allosteric pocket of hH_3_R, after ~ 6 ns of simulation, the hH_3_R started to undergo a conformational change especially in the intracellular part of the receptor. Here, a slight outward movement of TM VI was observed. Thus, the findings of the MD simulations support the experimental findings that a sodium ion, bound in its allosteric binding site stabilizes the inactive conformation of hH_3_R.

**Figure 6:**
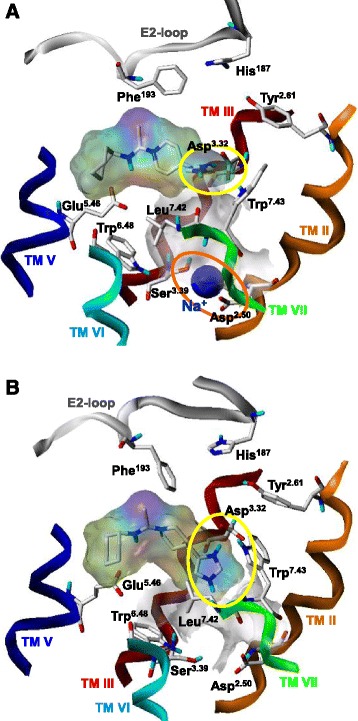
**Binding mode of thioperamide to the orthosteric binding site of hH**_**3**_ **R. A**, in presence of a sodium ion in the allosteric binding site. Yellow circle: interaction of the imidazole moiety of thioperamide with the highly conserved Asp^3.32^; orange circle: a sodium ion in its allosteric binding site, interacting with Ser^3.39^ and Asp^2.50^ **B**, in absence of a sodium ion in the allosteric binding site. Yellow circle: interaction of the imidazole moiety of thioperamide with the highly conserved Asp^3.32^, but compared to A) located downwards in direction to Asp^2.50^. Shown are snapshots after 5 ns of productive MD simulation.

In order to study the influence of a sodium ion in its allosteric binding site onto the binding mode of thioperamide in its orthosteric binding site, two different MD simulations were performed: On the one hand, one Na^+^ was placed into its allosteric site and one thioperamide was placed into its orthosteric binding pocket. On the other hand, one thioperamide was put into its orthosteric binding site, whereas no Na^+^ was located in the allosteric binding site. The MD simulations, performed under comparable conditions, revealed two different binding modes of thioperamide. In presence of a Na^+^ in the allosteric binding site, the imidazole moiety is located “above” the highly conserved Asp^3.32^ (Figure 6A) and the sodium ion remains stable in its allosteric site (Figure 6A). In contrast, in absence of the Na^+^ in the allosteric site, the imidazole moiety of thioperamide is located below the highly conserved Asp^3.32^, in direction to the highly conserved Asp^2.50^ (Figure 6B). As a consequence of the missing compensation of the negative charge of Asp^2.50^ by a sodium ion, the positively charged imidazole moiety of thioperamide is attracted by Asp^2.50^. However, in both cases, the thioperamide remains stable in its orthosteric binding pocket.

**Figure 7:**
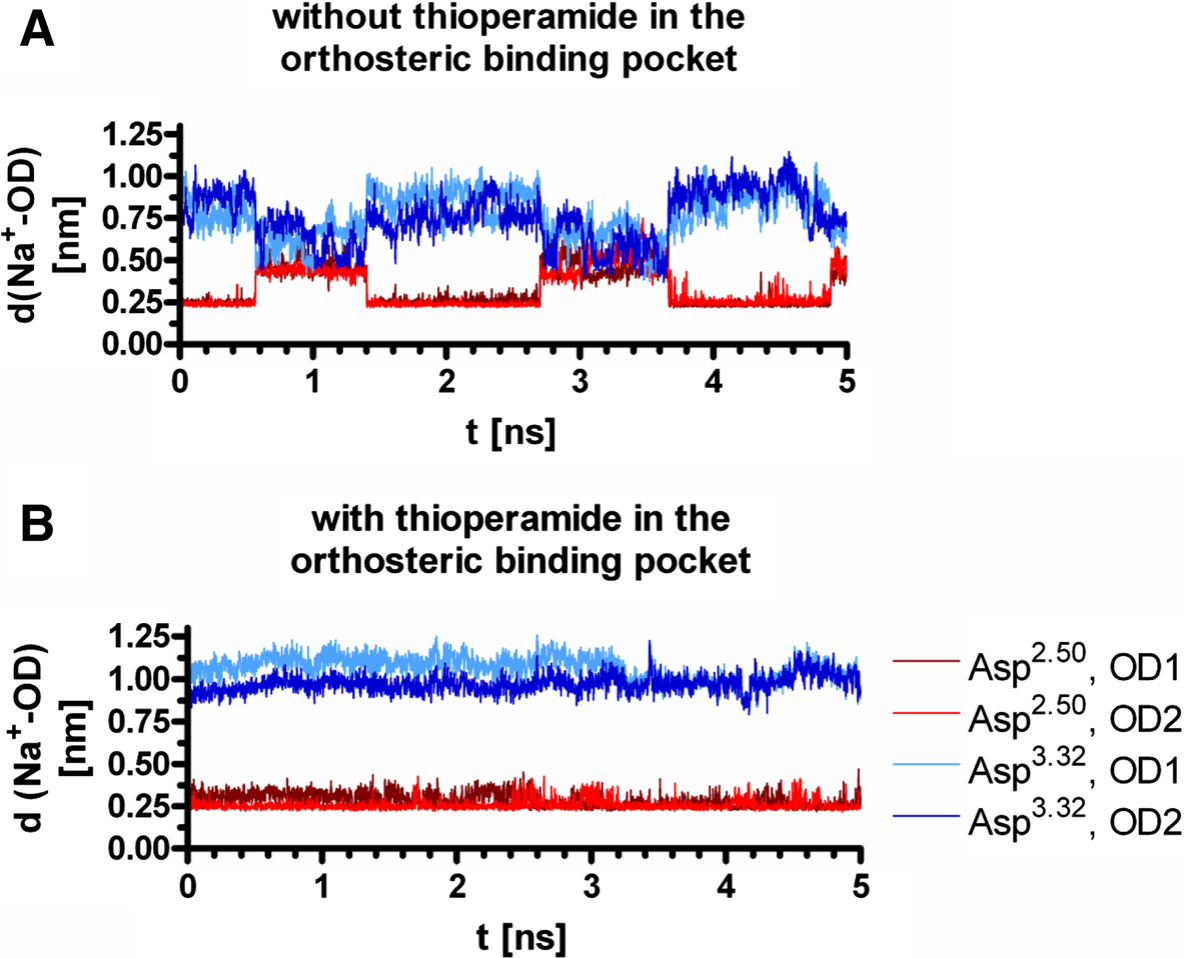
**Distance of the Na** ^**+**^ **in the allosteric binding site to Asp** ^**2.50**^ **and Asp** ^**3.32**^ **.** Shown are the data of representative 5 ns of MD simulation.

An analysis of the fluctuation of the sodium ion in the allosteric binding site in absence (Figure 7A) and presence (Figure 7B) of thioperamide in the orthosteric binding site is presented. In case that there is no ligand in the orthosteric binding pocket, the sodium ion shows a strong fluctuation between the Asp^2.50^ of the allosteric and Asp^3.32^ of the orthosteric site (Figure 7A). In case that the distance of the Na^+^ is with about 0.25 nm smallest to one of the carboxy oxygens (OD1 or OD2) of Asp^2.50^, the distance to one of the carboxy oxygens (OD1 or OD2) of Asp^3.32^ is about 0.87 nm (Figure 7A). In the other case, a distance of the Na^+^ to the Asp^2.50^ of about 0.4 nm was observed, whereas the distance of the Na^+^ to the Asp^3.32^ decreased to about 0.65 nm (Figure 7A). In the absence of a ligand, the sodium ion switches its position with a water molecule in the sodium binding channel. In contrast, if thioperamide is bound to the orthosteric binding site, the sodium ion remains very stable near to Asp^2.50^ and does not fluctuate between Asp^2.50^ and Asp^3.32^ (Figure 7B). Thus, the modelling data indicate that thioperamide in the orthosteric and Na^+^ in the allosteric site have a mutual influence to each other.

**Figure 8:**
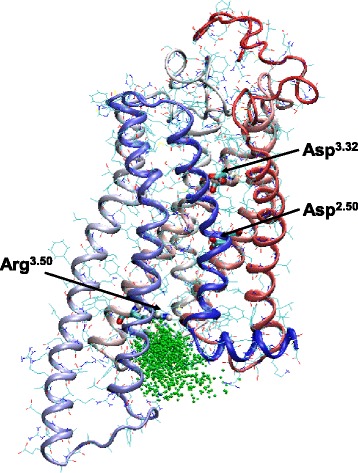
**Cl** ^**-**^ **binding site between the TM domains of the intracellular part of the hH**_**3**_ **R.** Shown are the positions of chloride ions (green spheres) accumulated over the whole simulation time.

An analysis of the positions of the chloride ions, present in the simulation box, during the simulation revealed no uniform distribution over the whole aqueous phase. Instead, a higher probability of chloride ions between the TM domains of the intracellular part of the hH_3_R relative to the remaining aqueous phase was observed (Figure 8). Although the increased probability of a chloride ion between the intracellular part of the TMs, a stable binding of one and the same Cl^-^ during more than 300 ps of the simulation was not observed. Because the intracellular part of the receptor is the target for the C-terminus of the Gα subunit, this observation may indicate that anions may have an influence onto the Gα-binding process and therefore onto GTP hydrolysis.

## Discussion

The results obtained in this study suggest that thioperamide, known as inverse agonist, not only binds into the inactive state hH_3_R, but also into the active hH_3_R. However, as suggested by the corresponding association constants, the binding of thioperamide to the inactive hH_3_R is preferred, compared to the binding to the active hH_3_R. Furthermore, the present results indicate that histamine, known as an agonist, not only binds to the active state hH_3_R, but also to the inactive hH_3_R. But as suggested by the corresponding association constants, the binding of histamine to the active hH_3_R is preferred, compared to the binding to the inactive hH_3_R. Thus, this kind of data analysis presented in this study allows to obtain information about association constants of a ligand to the inactive and active state of a GPCR separately.

In literature, the influence of different cations and anions onto the signalling of various GPCRs is discussed (Schetz and Sibley 2001; Swaminath et al 2002; Seifert and Wenzel-Seifert 2001; Schnell and Seifert 2010). At hH_3_R, the GTP hydrolysis in presence of 10 μM histamine or 10 μM thioperamide or the basal GTP hydrolysis without presence of a ligand was analyzed in dependence of the concentration of different monovalent salts, like LiCl, LiBr, LiJ, NaCl, NaBr, NaJ, KCl, KBr and KJ (Schnell and Seifert 2010). These data indicate that not only cations have an influence onto GPCR signalling, but also anions. Within the MD simulations at hH_3_R, chloride ions were observed more frequently at the intracellular side of the receptor. This is in very good accordance to the fact that, compared to the extracellular side of the hH_3_R, more positively charged amino acids are located at the intracellular side of the receptor. Although we could not detect a stable binding of the same chloride at the intracellular part of hH_3_R over several ns, we observed chloride ions binding for about 300 ps to positively charged amino acids of the hH_3_R, e.g. Arg^3.50^ (Figure 8), for several times during the whole simulation. This “sporadic” binding of a chloride ion may hinder the G protein to bind onto the active state hH_3_R, leading to a decreased basal activity with increasing concentration of chloride or other ions. Additionally, an effect of anions directly onto the G protein has to be considered (Higashijima et al. 1987). Relevant anion binding sites at G proteins can be identified within future studies by crystal structures or MD simulations. However, in order to obtain a deeper insight onto the influence of cations and anions onto GPCR signalling, more experimental studies, combined with modelling studies have to be performed. In this context it may be of interest to study the influence of monovalent salts onto the GPCR signalling of hH_4_R in more detail. Although it was shown that the hH_4_R is insensitive to sodium ions (Schneider and Seifert 2009), it may be useful to compare two different receptors, coupling to one and the same G protein in order to be able to separate between an effect of ions onto the receptor or onto the G protein.

**Figure 9:**
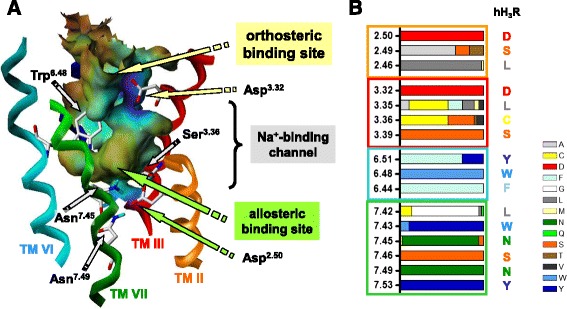
**Sodium binding channel of an aminergic GPCR. A**, Na^+^-binding channel of an aminergic GPCR with highly conserved amino acids. **B**, Distribution of amino acids being in direct contact to the sodium binding channel of all human aminergic GPCRs.

In general, functional data, e.g. obtained within the steady-state GTPase assay are analyzed by determination of the *pEC*_*50*_ value of a ligand. However, *pEC*_*50*_ values represent a complex quantity, consisting of distinct ligand- and/or receptor specific contributions, as shown above. Thus, a comprehensive explanation of *pEC*_*50*_ values on a molecular level with the help of computational methods may be a challenge. In contrast, if functional data will be analyzed using the equations mentioned above, distinct equilibrium constants, which can be related to results of molecular modelling studies, can be obtained. For example, using the present equations, it is possible to determine the binding constant for the sodium ion from the extracellular side, via the orthosteric binding site into its allosteric binding site. It may be suggested that there are no significant differences for the binding constant of the sodium ion from the extracellular side into the orthosteric binding pocket, because this process is mainly driven by an electrostatic attraction of the positively charged sodium ion and negatively charged amino acids in the orthosteric binding pocket, like the highly conserved Asp^3.32^. Thus, significant differences in the related constants *K*_*1*_ or *K*_*2*_ between sodium sensitive and sodium insensitive GPCRs are not expected. Of course, the binding of the sodium ion from the orthosteric to the allosteric binding site is also suggested to be driven by an electrostatic attraction between the sodium ion and the highly conserved Asp^2.50^ in the allosteric binding site. However, a comparison of all amino acids, forming the binding channel for the sodium ion from the orthosteric to the allosteric binding site, between the human aminergic GPCRs reveals distinct differences (Figure 9). Thus, it can be suggested that differences in amino acids between the human aminergic GPCRs within this channel may have a large influence onto sodium sensitivity, and consequently may have influence onto the constant *K*_*3*_, which corresponds to the transition of the sodium ion from its orthosteric to its allosteric binding site. Due to the differences in amino acids in direct neighbourhood to the sodium binding channel (Figure 9), it will be interesting to perform similar studies, as presented within this work, at other human aminergic GPCRs and to compare the resulting constants *K*_*3*_. This may give a more detailed insight onto the sodium sensitivity of GPCRs on a molecular level. Furthermore, the constants *K*_*6*_ (here describing the binding of thioperamide to the receptor with a sodium ion being in its allosteric binding pocket) and *K*_*9*_ (here describing the binding of histamine to the receptor with a sodium ion being in its allosteric binding pocket) are suggested to have an influence onto the sodium sensitivity of a GPCR. In general, if a sodium ion is bound in its allosteric binding site it has to be taken into account that this may have an influence onto the orthosteric ligand binding pocket, e.g. amino side chains being located in near neighbourhood to the allosteric and orthosteric binding site may change its conformation in dependence of absence or presence of a sodium ion in its allosteric site. Consequently this may have influence onto the binding properties of a ligand to its binding pocket. This hypothesis is supported by the MD simulations of thioperamide in the inactive hH_3_R (Figure 6). The results suggest that the binding mode of thioperamide is dependent of the absence or presence of a sodium ion in the allosteric pocket.

## Conclusion

In this study we developed a mathematical model to describe the sensitivity of GPCRs to sodium ions in presence or absence of a ligand. The excellent quality of the new mathematical model, consisting of a couple equilibrium constants, was shown by fitting experimental data obtained with the steady-state GTPase assay at hH_3_R. On the one hand, the new mathematical model allows a more detailed insight onto the ligand- and Na^+^ binding processes to a GPCR on a molecular level. On the other hand, the model may be extended to the quantitative description of arbitrary ligand-ion-receptor-binding processes.

## Abbreviations

Calc: Calculated
GPCR: G-protein coupled receptor
HIS: Histamine
THIO: Thioperamide
exp: Experimental
h: Human
H_3_R: Histamine H_3_ receptor
Δ_R_G^o^: Gibbs free energy of binding
TM: Transmembrane domain
